# Reliability of Kaiser Score in Assessing Additional Breast Lesions Identified on Staging MRI in Patients with Breast Cancer

**DOI:** 10.3390/diagnostics14161726

**Published:** 2024-08-08

**Authors:** Madiha Hijazi, Reve Chahine, Ghina Berjawi, Yara Jabbour, Tamara El Annan, Roy Ibrahim, Lara Nassar

**Affiliations:** 1 Department of Diagnostic Radiology, American University of Beirut Medical Center, Beirut 1107, Lebanon; mh354@aub.edu.lb (M.H.); chahinereve@gmail.com (R.C.); gb02@aub.edu.lb (G.B.); yj08@aub.edu.lb (Y.J.); te28@aub.edu.lb (T.E.A.); 2 Department of Diagnostic Radiology, Lebanese American University Medical Center, Beirut 1100, Lebanon; ibrahim_roy@hotmail.com

**Keywords:** Kaiser score, Tree flowchart, staging breast MRI, breast cancer, additional cancer

## Abstract

(1) Background: The Kaiser score is a user-friendly tool that evaluates lesions on breast MRI and has been studied in the general population and a few specific clinical scenarios. We aim to evaluate the performance of the Kaiser score in the characterization of additional lesions identified on staging breast MRI. (2) Methods: The Kaiser score of the biopsied additional lesions identified on staging MRI in recently diagnosed breast cancer patients was retrospectively determined. Statistical analysis was performed to evaluate the diagnostic capability of the Kaiser score and whether it is affected by different imaging and pathological parameters of the additional and the index lesion. (3) Results: Seventy-six patients with ninety-two MRI-detected lesions constitute the studied population. There was a statistically significant difference in the Kaiser score between benign and malignant lesions, irrespective of the pathology of the index cancer (*p* = 0.221) or the size and the imaging features of the additional lesion. Using a cutoff of 5 and above for suspicious lesions, biopsy could have been avoided in 34/92 lesions. (4) Conclusions: The Kaiser score can assist radiologists in the evaluation of additional MRI lesions identified in recently diagnosed breast cancer patients, thus decreasing the number of unneeded biopsies and delays in definitive surgical management.

## 1. Introduction

Breast cancer is the most common non-skin cancer among women, affecting nearly 2 million women every year, and accounting for the largest number of cancer-related deaths among women [1]. Compared to mammography, digital breast tomosynthesis and breast ultrasound, Magnetic Resonance Imaging (MRI) demonstrates higher sensitivity in detecting malignant lesions within the breast [2]. In women with recently diagnosed breast cancer, MRI has the potential to detect additional cancers in the ipsilateral and contralateral breast that may occasionally be more biologically aggressive than the index tumor [3,4]. A recent study by Nguyen et al. showed that most of the areas of non-mass enhancement in proximity to newly diagnosed breast cancer were malignant [5,6]. Although this has not yet been proven to have an actual impact on re-operation rate, disease-free survival, and overall survival [7], and has been associated with increased odds of receiving ipsilateral mastectomy and contralateral prophylactic mastectomy [8], MRI is being increasingly incorporated in the pre-operative staging of patients with recently diagnosed breast cancer [9].

The Breast Imaging Reporting and Data System (BI-RADS) is a standardized reporting system used to classify the findings observed in breast imaging studies to determine the level of suspicion for malignancy and the need for further imaging or intervention [10]. The numeric scoring ranges from 0 to 6 [11], and it undergoes periodic updates to incorporate the latest research findings and advancements in breast imaging technology [12].

However, although the BI-RADS MRI lexicon provides detailed descriptors to evaluate lesions, it does not specify the criteria used to assign a BI-RADS 3 or 4 score for each lesion, except when dealing with masses identified on MRI and exhibiting circumscribed oval margins without suspicious kinetics or a dominant T2 hypointense focus, which can be safely assigned a BI-RADS 3 score [13]. In several cases, assessing the need for biopsy in lesions becomes a matter of intuition and experience [14]. Therefore, there may be some degree of inter-observer variability in assigning MRI scoring systems affecting decision-making and patient management [8].

Moreover, significant overlaps exist in the imaging appearances of benign and malignant lesions. In patients undergoing staging breast MRI, the radiologists may tend to lower their threshold to recommend a biopsy when assessing an enhancing breast lesion due to the concern of missing an additional malignant lesion that may potentially impact the patient’s outcome. When added to the known high false positive rate of breast MRI [15], this may result in increased patient anxiety, added cost of further testing, and delays in definitive surgical management [8].

In 2013, a simple and time-efficient classification tree flowchart was introduced, that was later recognized as the Kaiser Score, which includes five diagnostic morphological and dynamic criteria, three ramifications, and eleven terminal nodes. Each combination of diagnostic criteria has a specific likelihood ratio that predicts the likelihood of a lesion to be benign or malignant. A score of 1–4 is usually associated with a benign lesion, whereas scores of 5 or above are more likely to represent suspicious lesions for which a biopsy is usually recommended. This score provides attending radiologists and residents with a tool that aids in differentiating breast lesions and directing management [16]. Kaiser score has improved diagnostic accuracy and lower discrepancies in lesion description rates among radiologists of different years of experience with higher specificity in inexperienced readers compared to BIRADS. This applies to mass and non-mass lesions [17].

Although this score was previously applied to MRI-only lesions with BIRADs 4 category and showed that it can obviate the need for biopsy in more than 25% of cases with a cutoff of 2 or less, representing exclusively benign lesions [18], the literature lacks, to our knowledge, its application on additional mammographically and sonographically occult lesions found on staging MRI of breast cancer patients.

This study aims to investigate the reliability of the Kaiser score in the evaluation of additional lesions identified on staging breast MRI in patients with recently diagnosed breast cancer.

## 2. Materials and Methods

### 2.1. Study Design

This is a retrospective single-centered study performed at the American University of Beirut Medical Center (AUBMC) in the Department of Diagnostic Radiology. After receiving approval from the Institutional Review Board (IRB), the hospital’s database was searched for all breast cancer women who have undergone staging breast MRI from January 2012 to January 2020 and in whom additional suspicious lesions were identified by MRI that were occult by mammography and breast ultrasound. Only those with a pathological diagnosis of the additional lesion (either through percutaneous biopsy or surgical excision) were included.

### 2.2. MRI Protocol

All patients are scanned prone in a 3.0 T magnet (Phillips Ingenia, Eindhoven, The Netherlands) and an InVivo 16-channel breast coil (Dunlee, Veenpluis 6, Best, The Netherlands). The protocol consisted of a localizer sequence followed by axial no SPAIR eTHRIVE (TR/TE 4.6/2.3, SNR 1, slice thickness 0.9 mm, matrix 400 × 400), T2 (TR/TE 5000/120, slice thickness 0.9 mm, isotropic reconstruction voxel 0.75/0.75/0.75), DWI (TR/TE 5000/80, slice thickness 2 mm, B values 0 and 800), and a non-contrast fat suppressed eTHRIVE sequence (TR/TE 4.8/2.1, slice thickness 0.9 mm, isotropic reconstruction voxel 0.75/0.75/0.75) followed by six dynamic sequences obtained at 90 sec intervals after IV administration of 0.1 mmol/kg of Dotarem. Coronal and sagittal reconstructions could be performed at the workstation as needed by the radiologist.

### 2.3. Data Collection

Four radiologists with different levels of experience in breast imaging examined all the MRI studies. They filled in the pathological diagnosis of the index cancer (Invasive Ductal Carcinoma (IDC), Invasive Lobular Carcinoma (ILC), or ductal carcinoma in situ (DCIS)), the imaging characteristics of the additional MRI detected lesion (number, size, type, shape, enhancement kinetics, and location), and its pathological diagnosis following biopsy. Kaiser score was assigned to the additional lesions using an online website (https://radiologie-weiterbildung.de/kaiser-score/ accessed on 20 May 2024 6:52 pm) that included mass and non-mass options. A training session was held before the initiation of imaging analysis. The radiologists were divided into two groups of two people who assigned a Kaiser score for each of the lesions. In cases of disagreement, the case was re-discussed among all four radiologists, and the final score was assigned after consensus. All radiologists were blinded to the histopathological findings of the additional lesions during the imaging analysis phase.

### 2.4. Statistical Analysis

For analysis purposes, all additional MRI-detected lesions with a pathology showing IDC, ILC, or DCIS were grouped as “cancer”. All other pathologies, including simple proliferative, multiple proliferative lesions, and lesions showing atypia were labeled “benign”.

In addition, the additional lesions were separated into two groups. Those with a Kaiser score of 1 to 4 were grouped, as those would usually be considered not suspicious, and another group was assigned to lesions with a Kaiser score of 5 or more in whom a tissue diagnosis would be warranted.

Data were analyzed using SPSS (Statistical Package for the Social Sciences) software version 27.

The association between Kaiser scores and breast cancer diagnosis was evaluated using the chi-square test and the logistic regression to calculate odds ratios (OR) and 95% confidence intervals (CI). Receiver Operating Characteristic (ROC) analysis was performed to assess the discriminatory ability of the Kaiser score, with the Area Under the Curve (AUC) providing a measure of the overall performance of the Kaiser score as a predictor.

Comparative analyses assessed differences in Kaiser scores across various categories using Mann–Whitney and Kruskal–Wallis tests. *p*-values less than 0.05 were considered statistically significant.

## 3. Results

**Figure 1:**
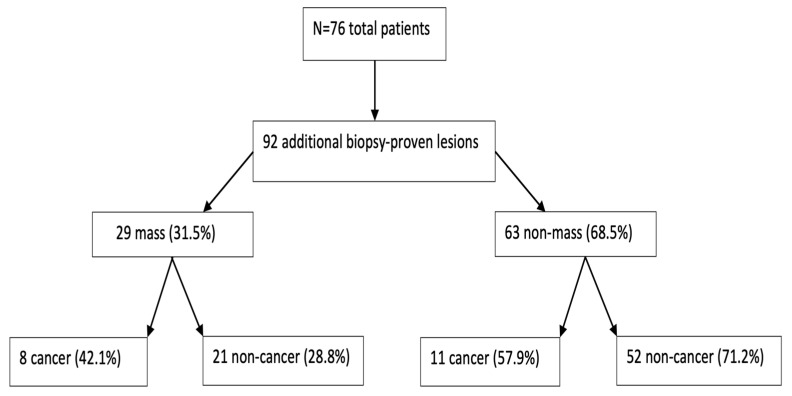
Distribution of MRI-detected additional lesions as cancer vs. noncancer.

A total of 76 patients were included in this study with a total number of 77 index lesions (56 masses (72.7%) and 21 non-mass lesions (27.3%)) and 92 additional MRI-detected lesions (29 masses (31.5%) and 63 non-mass lesions (68.5%)) (Figure 1). Of the additional lesions, 13 (14%) were in the same quadrant of the ipsilateral breast as the index cancer, 33 (35.5%) lesions were located within the ipsilateral breast but in different quadrants, and 46 (49.5%) lesions were located in the contralateral breast.

The statistical analysis of the Kaiser score assigned to additional lesions among breast cancer patients revealed a diverse distribution of scores. The most common Kaiser score observed is 6, accounting for 22.8% of the patients. This is followed by scores of 3 (14.1%), 7 (13.0%), and 1 (10.9%) (Table 1).

**Figure 2:**
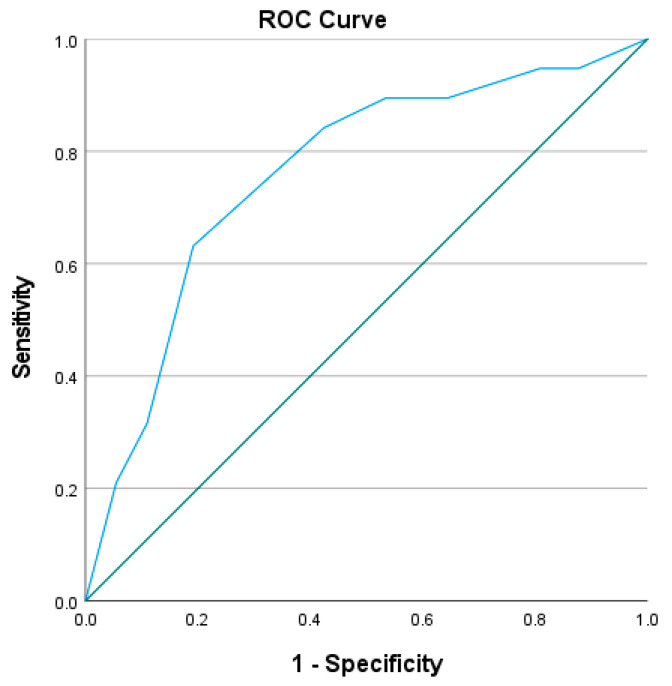
AUC for the Kaiser score as a predictor for breast cancer.

The ROC (Receiver Operating Characteristic) analysis for the Kaiser score as a predictor of malignancy yielded an AUC of 0.760 (95% [0.637–0.883] (Figure 2), indicating a diagnostic test.

**Figure 3:**
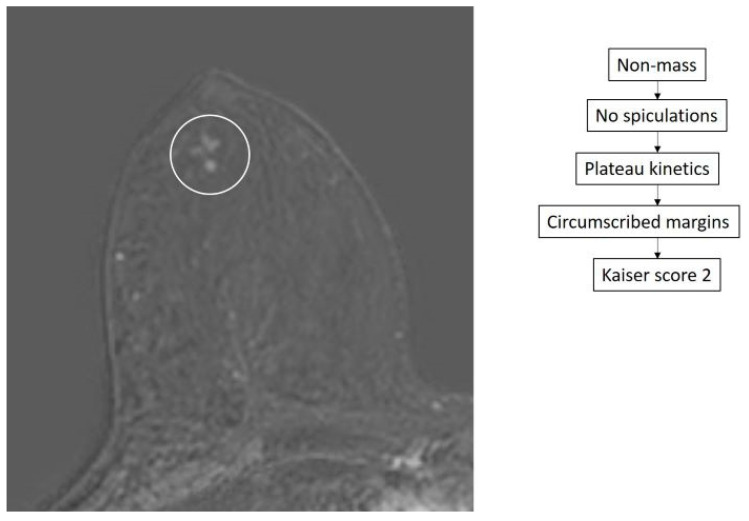
True negative case of a 47-year-old patient with a recent diagnosis of left breast cancer. Staging breast MRI shows clumped non-mass enhancement (circle) in the contralateral breast with a Kaiser score of 2. MRI guided biopsy was performed yielding proliferative fibrocystic changes.

**Figure 4:**
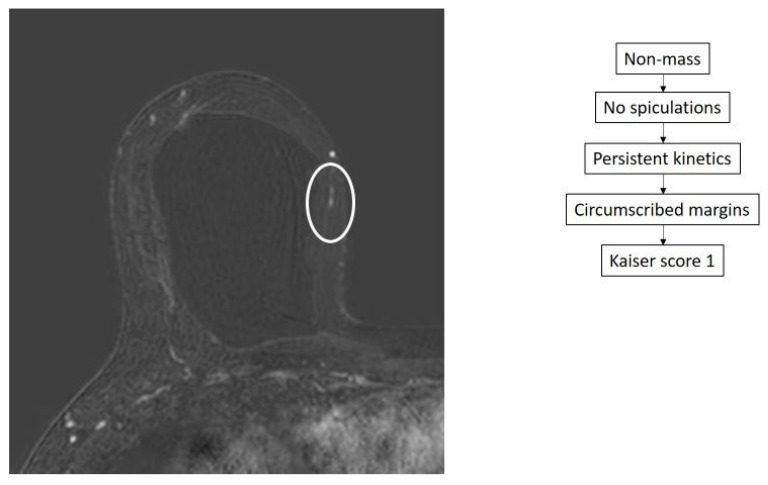
False negative case of a 55-year-old woman with recently diagnosed ductal carcinoma in situ (DCIS) in the lower outer quadrant of the right breast. An additional tiny linear non-mass enhancement is identified in the lower inner quadrant of the ipsilateral breast (circle), showing a Kaiser score of 1. MRI-guided biopsy was performed yielding DCIS.

Thirty-six lesions showed a Kaiser score between 1 and 4. Of these, 34 turned out benign by pathology (Figure 3) and 2 were malignant, consisting of 1 DCIS (Figure 4) and 1 invasive tubular carcinoma with DCIS. The characteristics of these two false negative results are displayed in Table 2.

**Figure 5:**
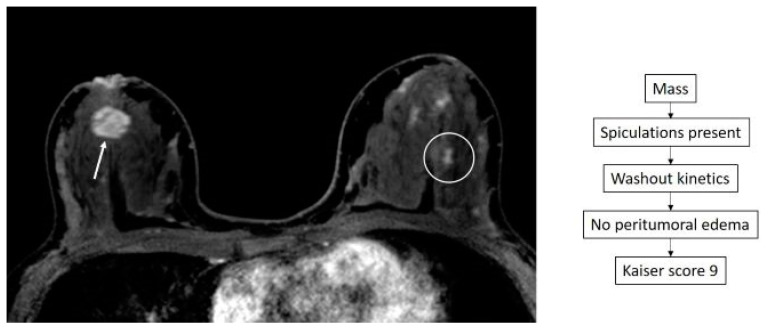
True positive case of a 37-year-old woman with recently diagnosed IDC in the central aspect of the right breast (arrow). An additional small irregular enhancing mass is identified in the lower outer quadrant of the contralateral breast (circle), showing a Kaiser score of 9. MRI-guided biopsy was performed yielding DCIS.

**Figure 6:**
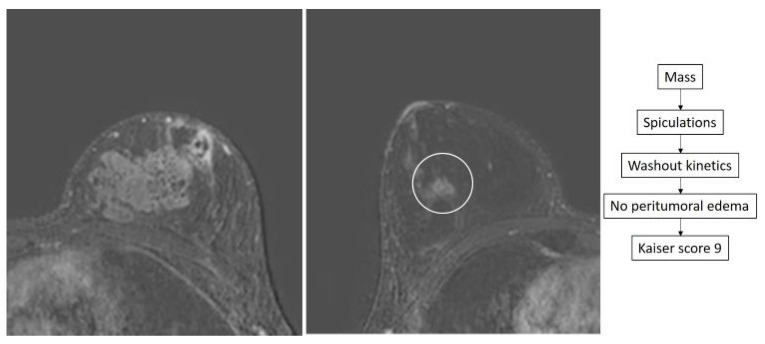
False positive case of a 25-year-old woman with recent diagnosis of locally advanced left breast cancer. Staging breast MRI shows an irregular mass (circle) in the contralateral breast showing some spiculations and exhibiting washout kinetics. The Kaiser score of this lesion was 9. A biopsy was performed showing intraductal papilloma and sclerosing adenosis.

Fifty-six lesions showed a Kaiser score of 5 and above; of these, 17 were malignant (Figure 5) and 39 were benign (Figure 6).

Using the cutoff set by the literature, results showed that patients with a Kaiser score of 5 and above had a higher incidence of cancer (PPV = 30.4%) compared to those with scores between 1 and 4 (5.6%), with a *p*-value of 0.004. This cut off shows a sensitivity of 89.5%, a specificity of 46.6%, and a negative predictive value of 94.4%. (Table 3).

There was no statistically significant difference in the performance of the Kaiser score when correlated to the pathological type of the index lesion, be it IDC, ILC, or DCIS (*p* = 0.221). Similarly, the discriminative capacity of the score was independent when correlated to the type of the lesion (mass v/s non mass *p* = 0.764), its shape (clumped, homogeneous, heterogeneous, rim, and linear *p* = 0.207), its initial enhancement characteristics (slow, medium, or rapid *p* = 0.877), its kinetic curve (persistent, plateau, or washout *p* = 0.601), its location (ipsilateral same quadrant *p* = 0.957, ipsilateral different quadrant *p* = 0.081, contralateral breast *p* = 0.087), and its size (*p* = 0.167) (Table 4). 

## 4. Discussion

Breast MRI is currently widely used by clinicians due to its high sensitivity and detailed tissue characterization for different indications, including staging in patients with biopsy-proven breast cancer [19]. As breast cancer can frequently be multifocal, multicentric, or bilateral, radiologists may tend to lower their threshold for recommending a biopsy in a patient undergoing staging breast MRI to avoid missing cancer in any subtle lesion. Since its introduction, the Kaiser score has been studied among different patient groups to classify lesions and assign a malignancy probability to aid clinicians of varying expertise levels in proper management, including equivocal lesions on diagnostic mammograms or contrast-enhanced mammography undergoing problem-solving MRI [20,21,22,23], BI-RADS 4 lesions identified on screening MRI [24], lesions presenting as non-mass enhancement [25], and other different indications [17,26], all showing high diagnostic performance of the Kaiser score. A variant has also been developed by Pötsch et al., who describe a modified version of the Kaiser score that can be applied to non-enhanced MRI examinations using morphological characteristics of the lesion on T2 in addition to its ADC value [27]. The Kaiser score has been compared to assessment using the BI-RADS descriptors [24] and to other scoring systems such as the Gottingen score [28] and has been shown to deliver a more reliable assessment of the lesions with a higher interobserver agreement.

This retrospective study evaluated the diagnostic ability of the Kaiser score in determining the malignity of additional lesions identified on staging MRI in patients with recently diagnosed breast cancer and yielded significant findings. Of the 56 lesions showing a Kaiser score of 5 and above, 30.4% were malignant. Moreover, 89.5% of the additional lesions that turned out to be malignant showed a Kaiser score of 5 or above. This indicates that higher Kaiser scores of lesions identified on breast MRI in patients undergoing staging breast MRI may be associated with a greater likelihood of a malignant diagnosis.

The AUC of 0.760 suggests that the Kaiser score has a fair discriminatory ability in distinguishing between benign and malignant lesions. Our study shows that at a score of 4 and below, the high NPV of 94.4% allows the radiologist to safely assign the additional lesion as benign without doubting the need for biopsy in this subcategory of patients. This finding follows other studies using the same cutoff in different populations of patients [20,21,24,29].

Two lesions were an exception and could have been missed as both turned out to be cancerous and were assigned Kaiser scores of 1 and 3 (Figure 3). While the NPV of the Kaiser score was very high, false negatives may still occur. This underscores the need to interpret the Kaiser score of the lesion in the context of the entire examination and clinical picture in order not to miss additional potentially malignant lesions.

Importantly, while the Kaiser score used at a cut-off of 5 and above to recommend a biopsy showed a very good discriminatory ability between benign and malignant lesions, this ability was independent of multiple parameters, including the type of the index cancer, as well as the location, size, and imaging characteristics of the additional lesion. This implies that when facing an additional lesion on a staging breast MRI, radiologists can confidently use the Kaiser score across all pathologies of biopsy-proven cancer, irrespective of the specific appearance of the additional lesions.

It is to be noted, however, that assigning a Kaiser score for a lesion includes evaluation of spiculations (root sign) in the setting of both mass and non-mass enhancement. While radiologists are familiar with evaluating spiculations in enhancing masses, doing the same for non-mass enhancement is not straightforward, especially for non-experienced readers. Indeed, the BI-RADS assessment of non-mass lesions that radiologists use describes the internal enhancement pattern as homogeneous, heterogeneous, clumped, and clustered ring appearance with no mention of spiculations. This may render an assessment of spiculation rather difficult in non-mass lesions [20,30]. To minimize this, it is better to assess spiculations on T2-weighted sequences, differentiate it from the margin criterion, and compare the findings with the original images in case of doubt in the subtraction series. It is advised to select the criterion as absent in case of high doubt when the radiologist subjectively fails to prove it is [31]. Similarly, evaluation of the computer-generated kinetic curve may be difficult in some cases, such as the false negative case displayed in Figure 3. However, this can be overcome by using a visual assessment of the kinetic curve as this has shown comparable results to computer-generated curves when used to assign a Kaiser score [32].

Despite the significant findings of this study, a few limitations must be mentioned. First, this study analyzed the Kaiser score in the specific subgroup of women with recently diagnosed breast cancer in whom an additional lesion was identified on staging breast MRI. Thus, our findings may not be generalizable to other population subgroups.

The study is also limited by its rather small sample size. Indeed, despite reviewing patients scanned between 2012 and 2020, we could only identify 92 additional biopsy proven lesions on staging breast MRI. A larger sample size would have resulted in better statistical power.

Moreover, while our study has demonstrated the use of the Kaiser score to be particularly reliable in excluding malignancy in lesions scoring 4 or less, the Kaiser score was assigned by consensus among the authors, thereby possibly affecting the reproducibility of this score among readers of different experience levels.

## 5. Conclusions

The use of the Kaiser score can assist radiologists in adequately categorizing additional lesions identified on staging breast MRI in women with newly diagnosed cancer, irrespective of the pathology of the index cancer and the imaging characteristics of the additional lesion. Its high negative predictive value could help radiologists avoid unnecessary biopsies and delays to definitive surgical management in lesions with a score of 1 to 4.

## Figures and Tables

**Table 1:** Distribution of Kaiser score values among the additional MRI-detected lesions.

	Additional Lesion		
Kaiser Score	Benign	Cancer	Total
1	9	1	10
2	5	0	5
3	12	1	13
4	8	0	8
5	8	1	9
6	17	4	21
7	6	6	12
8	4	2	6
9	4	4	8
10	0	0	0
11	0	0	0
Total	73	19	92

**Table 2:** Imaging and pathological characteristics of the two false negative lesions.

	Lesion 1	Lesion 2
Kaiser score	1	3
Pathology	DCIS	Invasive Tubular Carcinoma and DCIS
MRI type	Non-mass	Non-mass
MR shape	Linear	Clumped
Location with respect to the index cancer	Ipsilateral different quadrant	Ipsilateral different quadrant
Main suspicious feature	Additional unique enhancing lesion	Faint but asymmetrical enhancement compared to the contralateral side

**Table 3:** Representation of the Kaiser score with respect to the pathology of the additional lesion.

	Benign Lesion *n* (Percentage)	Cancer *n* (Percentage)	*p* Value *	OR	95% C.I. for EXP(B)
Kaiser score 1–4	34 (94.4%)	2 (5.6%)	0.004	7.410	1.5963–34.415
Kaiser score 5 and above	39 (69.6%)	17 (30.4%)			

* *p* value significant at less than 0.05.

**Table 4:** Correlation between the Kaiser score and the pathology of the index cancer, as well as the imaging features of the additional MRI-detected lesion.

	Kaiser Score		
	Score 1–4	Score 5 and above	*p* Value
**Index cancer**			
**Pathology**			0.221
DCIS	13 (52%)	12 (48%)	
IDC	16 (34%)	31 (66%)	
ILC	1 (20%)	4 (80%)	
**Additional lesion**			
**Size** **(mean in mm)**	12.6	16.8	0.167
**Location**			
Ipsilateral same quadrant			0.957
no	31 (39.2%)	48 (60.8%)	
yes	5 (38.5%)	8 (61.5%)	
Ipsilateral different quadrant			0.081
no	27 (45.8%)	32 (54.2%)	
yes	9 (27.3%)	24 (72.7%)	
Contralateral			0.087
no	14 (30.4%)	32 (69.6%)	
yes	22 (47.8%)	24 (52.2%)	
**Imaging appearance**			
Type			0.764
NME	24 (38.1%)	39 (61.9%)	
Mass	12 (41.4%)	17 (58.6%)	
Enhancement type			0.207
clumped	18 (42.9%)	24 (57.1%)	
heterogeneous	0 (0%)	4 (100%)	
homogeneous	11 (50%)	11 (50%)	
rim	0 (0%)	1 (100%)	
linear	5 (25%)	15 (75%)	
Initial enhancement			0.877
Slow	9 (36%)	16 (64%)	
Medium	4 (36.4%)	7 (63.6%)	
Rapid	22 (41.5%)	31 (58.5%)	
Delayed enhancement			0.601
Persistent	18 (45%)	22 (55%)	
Plateau	8 (33.3%)	16 (66.7%)	
Washout	9 (36%)	16 (64%)	

Analysis done using Kruskall–Wallis and Mann–Whitney tests.

## Data Availability

The raw data supporting the conclusions of this article will be made available by the authors upon request.
